# Modulation Effects of Curcumin on Erythrocyte Ion-Transporter Activity

**DOI:** 10.1155/2015/630246

**Published:** 2015-09-02

**Authors:** Prabhakar Singh, Syed Ibrahim Rizvi

**Affiliations:** Department of Biochemistry, University of Allahabad, Allahabad 211002, India

## Abstract

Curcumin ((1*E*,6*E*)-1,7-Bis(4-hydroxy-3-methoxyphenyl)-1,6-heptadiene-3,5-dione), the yellow biphenolic pigment isolated from turmeric (*Curcuma longa*), has various medicinal benefits through antioxidation, anti-inflammation, cardiovascular protection, immunomodulation, enhancing of the apoptotic process, and antiangiogenic property. We explored the effects of curcumin *in vitro* (10^−5^ M to 10^−8^ M) and *in vivo* (340 and 170 mg/kg b.w., oral) on Na^+^/K^+^ ATPase (NKA), Na^+^/H^+^ exchanger (NHE) activity, and membrane lipid hydroperoxides (ROOH) in control and experimental oxidative stress erythrocytes of Wistar rats. As a result, we found that curcumin potently modulated the membrane transporters activity with protecting membrane lipids against hydro-peroxidation in control as well as oxidatively challenged erythrocytes evidenced by stimulation of NKA, downregulation of NHE, and reduction of ROOH in the membrane. The observed results corroborate membrane transporters activity with susceptibility of erythrocyte membrane towards oxidative damage. Results explain the protective mechanism of curcumin against oxidative stress mediated impairment in ions-transporters activity and health beneficial effects.

## 1. Introduction

Curcumin, the biphenolic antioxidant isolated from turmeric (*Curcuma longa*) is reported to induce pleotropic health protective effects through its multitargeted and multifunctional bioactivities [1]. Health beneficial activities of curcumin include antioxidation, anti-inflammation, cardiovascular protection, immunomodulation, enhancing of the apoptotic process, and antiangiogenic properties [2]. Curcumin modulates cellular responses through regulating enzymes and ions-transporters activity directly or via altering membrane fluidity [3, 4]. However, the exact mechanism(s) which underlie the health-promoting effects of curcumin remain speculative.

Ion homeostasis plays a crucial role in maintaining ion gradients, cell volume, and action potential in nerve cell and smooth muscle for physiological processes [5]. Na^+^/H^+^ exchanger (NHE) is a ubiquitous electroneutral, amiloride-sensitive ion exchanger involved in regulation of alkalization and cellular acidosis by removal of hydrogen and influx of sodium in a 1 : 1 stoichiometric relationship [6]. NHE regulates cell volume, cellular growth and differentiation, and cell motility [7]. Similarly, ion-transporter, Na^+^/K^+^-ATPase (NKA), is a heterodimeric transmembrane ion pump acting as signal transducer, regulating the biochemical processes through ion homeostasis, neuronal signaling, muscle contraction, and substrate transportation [8]. The primary function of NKA is the regulation of ionic gradients across the cell membrane and osmotic equilibrium of the cell [9].

Mutation in the activity of membrane transport systems has been reported in various pathological conditions including diabetes, cancer, neurodisorders, CVD, and aging [10–13]. Impairment in transporters activity and deregulation of ions-homeostasis has been linked with membrane fluidity and susceptibility of membrane toward oxidative damage [14, 15]. Activity of superficially anchored or intersected enzymes, ions pump, and exchanger are modulated by minor changes in surrounding microenvironment; thus, modifications mediated through oxidative damage to membrane may be translated into effects on enzymes, ions pump, and exchanger activity [14, 16].

Previously, we reported that curcumin can modulate rat erythrocyte NKA activity* in vivo* [16], with* in silico* experiments pointing towards interaction of the compound with amino acids at the active site cavity of the enzyme [16]. The present study focuses on the dose-dependent effect of curcumin on NKA, NHE activity, and lipid hydroperoxidation in erythrocytes from normal and experimentally induced oxidative stressed Wistar rats.

## 2. Material and Methods

### 2.1. Chemicals and Instrument

Curcumin was purchased from Bio Basic Inc., Ontario, Canada (cat. number CB0346), and imidazole, Ouabain, Adenosine Tri-Phosphate (ATP), DIDS (4, 4-diisothiocyanatostilbene-2,2V-disulfonic acid), amiloride hydrochloride, and Bovine Serum Albumin (BSA) were purchased from Sigma Aldrich, India. Other chemicals of the highest purity were purchased from Merck, India, and HIMEDIA Labs, India. Digital pH meter Cyber scan PH 500 manufactured by Merck was used to measure the pH of solutions. Spectrophotometric measurements were performed on Shimadzu-UV-1800 (Japan) UV-VIS Spectrophotometer.

## 3. Experimental Study

### 3.1. Animal

Male albino rats (Wistar strain) of 6-7 months weighing between 150 and 200 g were purchased from CDRI-Lucknow, India. Animals were housed in polypropylene cages (6 rats per cage) at 24 ± 2°C and 12 h light : 12 h dark cycles. Animals were fed with standard pellet diet obtained from the Dayal Industries Limited, Lucknow, India, and had free access to drinking water. The protocol of the study was in conformity with the guidelines of the Institutional Ethical Committee of University of Allahabad.

Forty-two male Wistar rats were randomly divided into seven groups (six rats/group): Group (I), control, receiving no treatment/supplementation. In Group (II), experimental control, rats were supplemented with 0.9% NaCl solution through oral route. In Group (III), negative control, rats were treated with mercuric chloride (HgCl_2_, 5 mg/kg b.w., saline, intraperitoneal) to induce oxidative stress. In Group (IV), rats were treated with HgCl_2_ (5 mg/kg b.w., saline) and curcumin (340 mg/kg b.w., saline, oral). In Group [V], rats were treated with HgCl_2_ (5 mg/kg b.w., saline) and curcumin (170 mg/kg b.w., saline, oral). Group (VI) is curcumin treated group (340 mg/kg b.w., saline). Group (VII) is curcumin treated group (170 mg/kg b.w., saline). Curcumin treatment, oxidative stress induction, and sacrifices of rats were performed according to the method of Singh and Rizvi [17].

### 3.2. Preparation of Packed Red Blood Cells and Erythrocytes Membrane

The heparinized blood of rats was centrifuged at 800 ×g at 4°C for 10 min. After the removal of plasma, buffy coat, and upper 15% of the packed RBCs cells, the remaining RBCs were washed twice with 10 mM phosphate buffer saline pH 7.4. The erythrocytes membrane was isolated according to the method of Marchesi and Palade [18]. The erythrocyte membrane proteins were quantified according to the method of Lowry et al. [19].

### 3.3. *In Vitro* Experiments with Curcumin and Induction of Oxidative Stress

Oxidative stress was induced* in vitro* by incubating washed erythrocytes or erythrocytes membrane (1.5–2.0 mg of protein) with 10^−5^ M* tert*-butylhydroperoxide (*t*-BHP) for 30 min at 37°C; the effect of curcumin was evaluated by coincubating erythrocytes or erythrocytes membrane with* t*-BHP and curcumin for 30 min at 37°C with mild shaking as described previously [2].

### 3.4. Measurement of NHE Activity

NHE activity was calculated in terms of amiloride-sensitive H^+^-efflux from acid loaded cells as reported previously by Rizvi and Zaid [12]. The activity of the NHE modulated by polyphenols is extrapolated by difference in hydrogen efflux rates from acid loaded erythrocytes in the absence and presence of amiloride. Briefly, 0.2 mL of PRBCs was suspended in a 3.8 mL solution containing 150 mmol/L NaCl, 1 mmol/L KCl, 1 mmol/L MgCl_2_, and 10 mmol/L glucose and was incubated at 37°C for 5 min under continuous magnetic stirring. The erythrocytes suspension was brought to pH 6.35–6.45 within 10 min using 0.2 mol/L HCl solutions in 150 mmol/L NaCl. Now, DIDS was added to the medium (0.2 mmol/L final concentration) and the pH value was brought to 7.95–8.00 using 0.05 mol/L NaOH solution in 150 mmol/L NaCl. In a parallel experiment, inhibitor amiloride (0.5 mmol/L final concentration) was added with DIDS. Thereafter, the first minute proton efflux was recorded. The rate of NHE (proton efflux *μ*mol/L PRBC/h at 37°C) activity derives from the difference in rates of medium acidification in the absence (ΔpH_1_) and presence (ΔpH_2_) of amiloride, corrected by the buffer capacity of the incubation medium (b), erythrocytes volume in the suspension, and the incubation time.* In vitro* experiments were carried out by adding curcumin to the erythrocytes suspension and incubating for 30 min at 37°C before assay.

### 3.5. Measurement of NKA Activity

NKA activity was measured according to the methods of Suhail and Rizvi [20]. The final assay mixture contained 0.5–1.2 mg membrane protein/mL, 20 mmol/L KCl, 140 mmol/L NaCl, 3 mmol/L MgCl_2_, and 30 mmol/L imidazole (pH 7.24), with or without 5 × 10^−4^ mol/L Ouabain and 6 mmol/L ATP. Assay mixture was incubated for 30 min at 37°C and the reaction in assay mixture was stopped by the addition of 3.5 mL of solution A (0.5% ammonium molybdate, 0.5 mol/L H_2_SO_4_, and 2% SDS). *K*_*m*_ and *V*_max_ value of NKA were measured in the presence of 20 mM, 10 mM, 7 mM, 5 mM, and 3 mM of substrate ATP. The amount of liberated phosphate (pi) was estimated according to the method of Fiske and Subbarow [21].* In vitro* experiment was carried out by adding curcumin (final concentration 10^−5^ M to 10^−8^ M) to the assay mixture and incubating for 30 min at 37°C prior to enzyme assay. NKA activity was expressed as nmol pi released/mg RBCs membrane protein per hour at 37°C.

### 3.6. Determination of ROOH

Lipid hydroperoxides (ROOH) value in erythrocyte membrane was measured according to the methods of Mehdi et al. [22]. For measuring ROOH, 200 *μ*L of EDTA (Ethylenediaminetetraacetic Acid) free erythrocytes membrane (0.8 to 1.0 mg protein/mL) was taken in duplicate tubes. In one of the tubes, 20 *μ*L triphenylphosphine (methanolic) was added, and in another, 20 *μ*L of methanol was added. The tubes were vortex-mixed every 10 min and incubated at room temperature in the dark for 30 min. After completion of incubation period, 1800 *μ*L of FOX solution (solution A: 4.4 mM butylated hydroxytoluene in methanol, solution B: 1 mM xylenol orange and 2.5 mM ammonium ferrous sulphate dissolved in sulfuric acid 250 mM. A working solution was prepared by mixing A and B solutions at a proportion of 9 : 1 resp.) was added in both tubes. The samples were again incubated for 1 hour at room temperature in the dark with vortex-mixing at every 10 min. After second incubation, samples were centrifuged at 16,000 g for 7 minutes and absorbance of the supernatants was measured at 560 nm. The absorbance of the samples treated with TPP was subtracted from nontreated samples to calculate the concentration of ROOH. Results were calculated with the aid of a calibration curve using hydrogen peroxide in the range 1–10 *μ*M. ROOH is expressed as nmol/mg RBCs membrane protein.

### 3.7. Statistical Analysis

Statistical analysis was performed by Graph Pad Prism 5 version 5.01 (Graphpad Software Inc., San Diego, California, USA). One way analysis of variance (ANOVA) was performed for multiple comparisons. *p* values were evaluated by two tailed method. All the values with *p* < 0.05 were considered as statistically significant. Values are represented as ±SD in graphs. Significance from experimental control is represented by star (*∗*) and from negative control (HgCl_2_) is represented by hatch (#). Kinetic evaluation was done according to the methods of Singh and Rizvi [14].

## 4. Results

**Figure 1:**
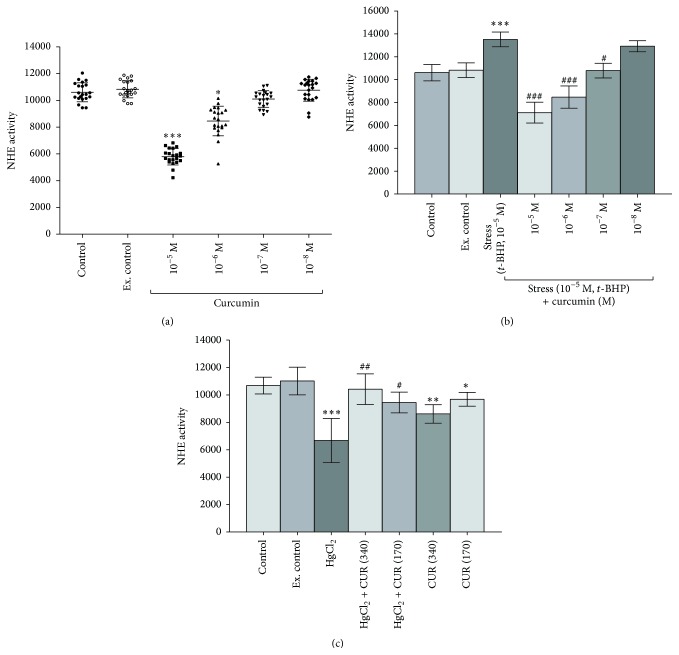
*In vitro* dose-dependent effect of curcumin (10^−5^ M to 10^−8^ M) on Wistar rats erythrocytes NHE activity. (a) Healthy erythrocytes. (b) Stressed (*t-*BHP, 10^−5^ M) erythrocytes. (c)* In vivo* effect of curcumin (340 mg/kg b.w. and 170 mg/kg b.w. oral) on healthy and oxidatively stressed (HgCl_2_, 5 mg/kg b.w.) Wistar rats erythrocytes NHE activity. NHE activity is expressed as proton efflux *μ*mol/L PRBC/h at 37°C. Values are mean ± SD.

*In vitro* treatment of curcumin (10^−5^ M to 10^−8^ M) to rat erythrocytes resulted in concentration dependent inhibition of NHE activity. Maximum NHE inhibition (*p* < 0.001) was observed at 10^−5^ M which decreased gradually on lowering the concentration of curcumin (Figure 1(a)). Inducing oxidative stress by* t*-BHP (10^−5^ M) caused activation (*p* < 0.001) of NHE activity which was significantly (*p* < 0.001) decreased by curcumin in a dose-dependent manner. However, no significant protection against* t*-BHP induced activation of NHE was observed at 10^−8^ M curcumin (Figure 1(b)).

*In vivo* induction of oxidative stress in rats by HgCl_2_ (5 mg/kg b.w, i.p. saline) significantly (*p* < 0.001) decreased the NHE activity which was reversed by oral supplementation of curcumin (340 and 170 mg/kg b.w.). Supplementation of curcumin to control rats also caused concentration dependent downregulation of NHE activity (Figure 1(c)).

**Figure 2:**
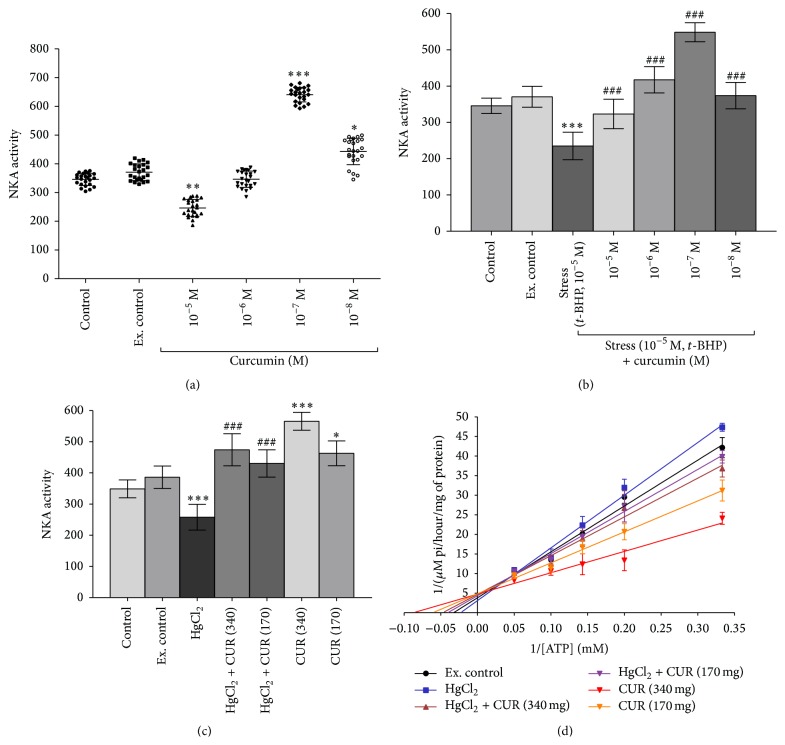
*In vitro* dose-dependent effect of curcumin (10^−5 ^M to 10^−8 ^M) on Wistar rats erythrocytes NKA activity (Ouabain-sensitive). (a) Healthy erythrocytes. (b) Stressed (*t-*BHP, 10^−5 ^M) erythrocytes. (c)* In vivo* effect of curcumin (340 mg/kg b.w. and 170 mg/kg b.w. oral) on healthy and oxidatively stressed (HgCl_2_, 5 mg/kg b.w.) Wistar rats erythrocytes membrane NKA activity (Ouabain-sensitive). NKA activity expressed in terms of nmol pi released/h/mg membrane protein at 37°C. Values are mean ± SD. (d)* In vivo* NKA activity (Lineweaver-Burk plot) in control (black circle), HgCl_2_ (blue square), HgCl_2 _+ CUR (340) (dark red triangle), HgCl_2 _+ CUR (170) (purple triangle), CUR (340) (red triangle), and CUR (170) (orange triangle) treated rat erythrocytes membrane is plotted as a function of ATP concentration. *V*_max_ of NKA activity is expressed as *μ*mol pi released/h/mg membrane protein at 37°C. The intercept on the ordinate is equal to 1/*V*_max_ and the intercept on the abscissa is equal to −1/*K*_*m*_. Values in graph represent control (*K*_*m*_ = 30.62 mM, *V*_max_ = 0.2615), HgCl_2_ (*K*_*m*_ = 43.30 mM, *V*_max_ = 0.3225), HgCl_2_ + CUR (340) (*K*_*m*_ = 21.06 mM, *V*_max_ = 0.2130), HgCl_2_ + CUR (170) (*K*_*m*_ = 24.66 mM, *V*_max_ = 0.2297), CUR (340) (*K*_*m*_ = 11.44 mM, *V*_max_ = 0.21004), and CUR (170) (*K*_*m*_ = 16.37 mM, *V*_max_ = 0.2071). The method of least squares has been used to fit the data to the Lineweaver-Burk plot. Values represent mean ± SD and *r* ^2^ = 0.9710 (control), *r* ^2^ = 0.9751 (HgCl_2_), *r* ^2^ = 0.9468 (HgCl_2_ + CUR-340), *r* ^2^ = 0.9616 (HgCl_2_ + CUR-170), *r* ^2^ = 0.8794 (CUR-340), and *r* ^2^ = 0.9564 (CUR-170) of linear regression. *V*_max_ of NKA activity expressed in terms of nmol pi released/h/mg membrane protein at 37°C. Values are mean ± SD.

*In vitro* treatment of rat erythrocytes membrane with curcumin (10^−5^ M to 10^−8^ M) shows biphasic effects on NKA activity. A significant (*p* < 0.001) NKA activity inhibition response was observed at 10^−5^ M curcumin. No significant changes in NKA activity were observed at 10^−6^ M curucmin. However, a significantly (*p* < 0.01) increased NKA activity was observed at 10^−7^ and 10^−8^ M. No significant inhibitory response was observed at 10^−5^ M (Figure 2(a)). Inducing oxidative stress by* t*-BHP (10^−5^ M) caused significant (*p* < 0.001) inhibition of NKA activity, which was reversed (*p* < 0.001) in the presence of curcumin 10^−5^ to 10^−8^ M (Figure 2(b)).

*In vivo* inducing oxidative stress to rats by HgCl_2_ (5 mg/kg b.w, i.p. saline) decreased the NKA activity (*p* < 0.001). Oral supplementation of curcumin (340 and 170 mg/kg b.w.) significantly (*p* < 0.001) reversed the effects. In control rats, curcumin also caused significant (*p* < 0.05) activation of NKA (Figure 2(c)).

Lineweaver-Burk plot of NKA activity with ATP as substrate (20 mM, 10 mM, 7 mM, 5 mM, and 3 mM) shows that *K*_*m*_ of NKA was significantly (*p* < 0.001) increased in oxidative stress condition *K*_*m*_ = 30.62 mM to *K*_*m*_ = 43.30 mM. Curcumin supplementation (340 mg/kg b.w. and 170 mg/kg b.w) to oxidative stress induced rats reduced *K*_*m*_ from 43.30 mM to 21.06 mM and 24.66 mM, respectively. In addition, supplementation of curcumin 340 mg/kg b.w. and 170 mg/kg b.w. to experimental control rats caused significant reduction of *K*_*m*_ value from 30.62 mM to 11.44 mM and 16.37 mM, respectively (Figure 2(d)).

**Figure 3:**
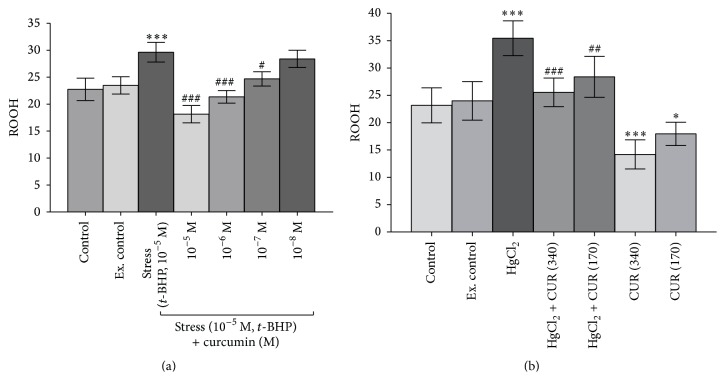
(a)* In vitro* dose-dependent effect of curcumin (10^−5^ M to 10^−8^ M) on lipid hydroperoxides (ROOH) in Wistar rat erythrocyte subjected to oxidative stress (*t-*BHP, 10^−5^ M). (b)* In vivo* effect of curcumin (340 mg/kg b.w. and 170 mg/kg b.w. oral) on lipid hydroperoxides (ROOH) in Wistar rats subjected to oxidative stressed (HgCl_2_, 5 mg/kg b.w.). ROOH value expressed in terms of nmole/mg erythrocytes membrane protein. Values are mean ± SD.

*In vitro*, curcumin (10^−5^ M to 10^−8^ M) was found to protect the membrane lipid constituent of rat erythrocytes against oxidative stress* t*-BHP (10^−5^ M). Inducing oxidative stress by* t*-BHP (10^−5^ M) caused upregulation of lipid hydroperoxides (ROOH) formation which was significantly (*p* < 0.001) decreased by curcumin (Figure 3(a)). Similarly,* in vivo *oral supplementation of curcumin (340 and 170 mg/kg b.w.) significantly (*p* < 0.001) reduced the ROOH content in erythrocytes membrane of experimental control rats as well as oxidatively challenged rats (HgCl_2_, 5 mg/kg b.w, i.p. saline) (Figure 3(b)).

## 5. Discussion

Several stimuli regulate the activity of NHE through the interaction with membrane receptors coupled with tyrosine kinases, G-proteins, or integrins [23]. The role of activated NHE activity in erythrocytes has been reported during aging, diabetes, and other age-related diseases. We observed that* in vitro* curcumin treatment reduced the NHE activity in healthy erythrocytes of rat (Figure 1(a)).* In vitro* presence of oxidative stress (*t*-BHP, 10^−5 ^M) has been reported to induce NHE activity [5]. Our results show a similar result for NHE activity in erythrocytes subjected to oxidative stress (*t*-BHP, 10^−5^ M). Curcumin has been observed to mitigate oxidative stress and reverse the NHE activity in erythrocytes (Figure 1(b)).

Oxidative stress at higher concentration has been found to reduce the NHE activity in biliary epithelial cancer cell line (Mz-Cha-1) and associated with glutathione redox system [24]. Curcumin increased GSH concentration against oxidative stress in human erythrocytes [2]. We found* in vivo* induction of oxidative stress (HgCl_2_, 5 mg/kg b.w.) reduced the NHE activity, but oral supplementation of curcumin mitigated the oxidative stress and reduced the NHE activity in erythrocytes (Figure 1(c)).

Hyperactivity of NHE has been reported to be directly associated with uncontrolled proliferation of neoplastic cells and motility and invasion of cancer cells derived from various tissues [25]. Increased or overexpressed NHE activity induced cytoplasm alkalization and vascular disorders as cardiac injury and arrhythmias [26]. NHE inhibition is emerging as an effective strategy to minimize invasiveness of the neoplastic breast cancer and myocardial remodeling as well as improve efficacy of resuscitation following cardiac arrest. Downregulation of NHE activity has been implicated in tumor cell death through cell growth arrest, acidification of the intracellular milieu, and sensitization to death triggers [27–29]. NHE inhibitors have also been found to protect mitochondrial integrity with reduction in ROS synthesis [30–32].

NKA activity synchronized with fluidity of membrane and by various drugs affecting the membrane fluidity [3, 33]. Oxidative stress and/or ROS modifies the lipid environment as well as fluidity of membrane and is known to inhibit NKA activity of erythrocytes [34]. Curcumin intercepts and localizes itself between polar head and nonpolar tail of lipid molecule in plasma membrane and changes the fluidity and thickness of membrane [35]. Curcumin modulated the NKA activity in erythrocytes of healthy human and rats by interacting with amino acids Thr, Glu, Val, Arg, Tyr, Gly, Ser, Ile, Phe, Tyr, and Ile at the active site cavity of Na^+^/K^+^-ATPase *α* unit [16]. Here, we found that* in vitro* curcumin modulated the NKA activity in a biphasic manner in healthy rat erythrocytes (Figure 2(a)). In addition, curcumin also protects the NKA activity against oxidative stress in erythrocytes of rat (Figure 2(b)). Upregulation of NKA activity by curcumin has also been observed* in vivo* against oxidative stress (Figure 2(c)). Kinetic evaluation revealed that oxidative stress increased *K*_*m*_ of NKA. Curcumin presence reversed the effects of oxidative stress as evidenced by increased NKA activity and decreased *K*_*m*_ (Figure 2(d)). Curcumin treatment effects are in agreement with the finding of Kaul and Krishnakanth (1994) that curcumin treatment reduced the *K*_*m*_ value of NKA enzyme. They found that curcumin supplementation to rats against retinol deficiency increased the NKA activity in the brain microsomal membrane through reducing *K*_*m*_ and *V*_max_ [36]. Curcumin has been found to interact with transmembrane domain(s) of the *α*-subunit of NKA protein to modulate transporter activity [37]. NKA inhibition decreases the norepinephrine dopamine and serotonin (5-HT) uptake with increasing acetylcholine release and hence alters neuronal firing and impairs spatial and other forms of learning [38]. An altered NKA activity is reported during late complications of diabetes mellitus, nephropathy, neuropathy, and retinopathy, and in the development of diabetic vascular complications [20, 34, 39].

Lipid peroxidation is highly destructive process in which unsaturated fatty acids of membrane are degraded to small and highly reactive conjugated dienes, thiobarbituric acid-reactive substances (TBARs), and lipid hydroperoxides (ROOH) [40], accompanying pathological and toxicological events* in vivo* [41]. Curcumin has hydrogen-donating antioxidant potential against oxidative stress inhibiting lipid peroxidation and augmenting the antioxidant potential in cell [1]. Previously, we have reported that curcumin reduced the malondialdehyde formation in human erythrocytes against oxidative stress [2]. Here, we observe that curcumin reduced the ROOH formation in erythrocytes membrane against oxidative stress* in vitro* as well* in vivo *(Figures 3(a) and 3(b)). ROOH level provides an index of membrane lipid constituent susceptibility towards oxidative damage and thus membrane integrity. Increased ROOH level in the blood has been accounted as a consistent mark of increased oxidative stress in tissues like skeletal muscle, liver, and heart [42].

## 6. Conclusion

On the basis of results of the study, we conclude that a strong correlation exists between the effects of curcumin on redox status and NKA activity and/or perhaps NKA expression level. Downregulation of NHE activity, modulation of NKA activity, and inhibition of ROOH synthesis in erythrocytes membrane by curcumin under actual or simulated conditions of oxidative stress emphasize the important role of curcumin. Our findings provide a mechanistic explanation to some of the pleiotropic biological effects of curcumin.

## Abbreviation

NHE: Sodium-Hydrogen Exchanger
NKA: Na^+^/K^+^-ATPase
ROOH: Lipid hydroperoxides
*t*-BHP: * tert*-butylhydroperoxide
PRBCs: Packed Red Blood Cells.
